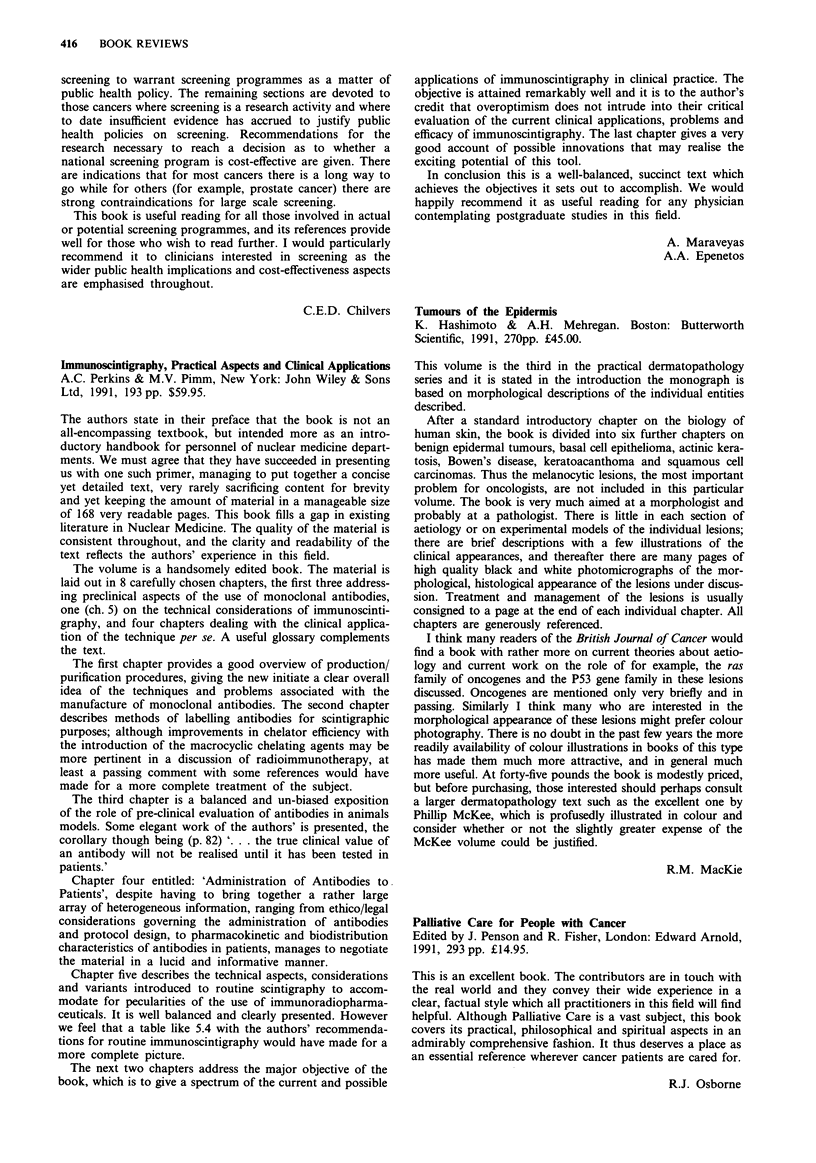# Tumours of the Epidermis

**Published:** 1992-08

**Authors:** R.M. MacKie


**Tumours of the Epidermis**
K. Hashimoto & A.H. Mehregan. Boston: Butterworth Scientific, 1991, 270pp. £45.00.

This volume is the third in the practical dermatopathology series and it is stated in the introduction the monograph is based on morphological descriptions of the individual entities described.

After a standard introductory chapter on the biology of human skin, the book is divided into six further chapters on benign epidermal tumours, basal cell epithelioma, actinic keratosis, Bowen's disease, keratoacanthoma and squamous cell carcinomas. Thus the melanocytic lesions, the most important problem for oncologists, are not included in this particular volume. The book is very much aimed at a morphologist and probably at a pathologist. There is little in each section of aetiology or on experimental models of the individual lesions; there are brief descriptions with a few illustrations of the clinical appearances, and thereafter there are many pages of high quality black and white photomicrographs of the morphological, histological appearance of the lesions under discussion. Treatment and management of the lesions is usually consigned to a page at the end of each individual chapter. All chapters are generously referenced.

I think many readers of the *British Journal of Cancer* would find a book with rather more on current theories about aetiology and current work on the role of for example, the *ras* family of oncogenes and the P53 gene family in these lesions discussed. Oncogenes are mentioned only very briefly and in passing. Similarly I think many who are interested in the morphological appearance of these lesions might prefer colour photography. There is no doubt in the past few years the more readily availability of colour illustrations in books of this type has made them much more attractive, and in general much more useful. At forty-five pounds the book is modestly priced, but before purchasing, those interested should perhaps consult a larger dermatopathology text such as the excellent one by Phillip McKee, which is profusedly illustrated in colour and consider whether or not the slightly greater expense of the McKee volume could be justified.

R.M. MacKie